# Responses to fertility treatment among patients with cancer: a retrospective cohort study

**DOI:** 10.1186/s40738-018-0048-2

**Published:** 2018-04-17

**Authors:** A. V. Dolinko, L. V. Farland, S. A. Missmer, S. S. Srouji, C. Racowsky, E. S. Ginsburg

**Affiliations:** 1 Department of Obstetrics, Gynecology, and Reproductive Biology, Division of Reproductive Endocrinology and Infertility, Brigham and Women’s Hospital and Harvard Medical School, 75 Francis Street, ASB I-3, Boston, MA 02115 USA; 2 Channing Division of Network Medicine, Department of Medicine, Brigham and Women’s Hospital and Harvard Medical School, 181 Longwood Avenue, Boston, MA 02115 USA; 3 Department of Epidemiology, Harvard T.H. Chan School of Public Health, 677 Huntington Ave, Boston, MA 02115 USA; 4 Present address: Department of Obstetrics and Gynecology, Women & Infants Hospital, 101 Dudley St., Providence, RI 02905 USA

**Keywords:** Cancer, Oncofertility, Fertility preservation, Assisted reproductive technology

## Abstract

**Background:**

Cancer treatments have significant negative impacts on female fertility, but the impact of cancer itself on fertility remains to be clarified. While some studies have shown that compared with healthy women, those with cancer require higher doses of gonadotropins resulting in decreased oocyte yields, others have shown comparable oocyte yields between the two groups. The purpose of this study is to evaluate whether there is an association between any cancer and/or type of cancer, and response to ovarian stimulation for egg and embryo banking.

**Methods:**

In this retrospective cohort study, ovarian stimulation cycles performed from June 2007 through October 2014 at a single academic medical center were reviewed to identify those undertaken for women with cancer undergoing fertility preservation (*n* = 147) or women with no cancer undergoing their first cycle due to male factor infertility (*n* = 664). Of the 147 women undergoing fertility preservation, 105 had local cancer (Stage I-III solid malignancies) and 42 had systemic cancer (hematologic or Stage IV solid malignancies). Response to ovarian stimulation was compared among these two groups and women with no cancer.

**Results:**

Adjusting for age and BMI, women with systemic cancer had lower baseline antral follicle counts (AFC) than women with no cancer or local cancer. Women with systemic cancer required higher doses of FSH than women with no cancer or local cancer, and they had higher oocyte to AFC ratios than women with no cancer or local cancer, but greater odds of cycle cancellation as compared to women with no cancer or local cancer. No significant differences were observed among the three groups for duration of stimulation, number of oocytes and mature oocytes retrieved, or number of embryos created.

**Conclusions:**

Women with cancer achieve similar oocyte and embryo yields as women with no cancer, although those with systemic cancer require higher FSH doses and are at greater risk of cycle cancellation.

## Background

Approximately 47,500 women between the ages of 15 and 39 were diagnosed with cancer in the United States in 2012 [1]. As new treatment options have developed, 5-year survival rates for female cancer patients have improved and an increasing number of women are looking forward to resuming life after treatment [2]. However, the available therapies are often gonadotoxic and threaten women with loss of fertility. While it is well established that cancer treatments have a significant negative impact on female fertility, it is still under debate whether the presence of cancer has a detrimental impact on ovarian function and/or response to controlled ovarian stimulation.

Over the past few decades, several fertility preservation (FP) options have been developed, including the cryopreservation of oocytes and embryos. While embryo storage is a well-established option, mature oocyte cryopreservation has only recently been upgraded in the United States from an experimental strategy to an accepted therapeutic option [3, 4]. We have previously shown that women with cancer who underwent FP in our program required a higher total dose of gonadotropins and produced a lower number of mature oocytes than non-oncologic in vitro fertilization (IVF) control patients, although the total number of oocytes retrieved and the number of embryos produced was not significantly different [5]. Although both embryo and oocyte cryopreservation are now accepted and recommended techniques for FP, data regarding outcomes with thawed embryo transfer or thawed oocyte fertilization and subsequent embryo transfer in cancer patients is limited.

The aims of this study were to expand our knowledge of possible associations between the type of malignancy on ovarian function, ovarian stimulation, and pregnancy rates following thawed oocyte/embryo transfer. Our goal is to provide more robust information for providers when counseling patients diagnosed with malignancies about the efficacy of FP.

## Methods

### Selection criteria

Between July 9, 2007 and October 31, 2014, 13,221 consecutive ovarian stimulation cycles, with or without intracytoplasmic sperm injection (ICSI), were performed at our institution. All cycle data were retrospectively reviewed to identify women undergoing assisted reproduction to cryopreserve eggs and/or embryos for the purposes of FP in the setting of a cancer diagnosis (*n* = 153 cycles). Of these cycles, four were excluded because they were performed in women who had undergone prior ovarian stimulation cycles. Two cycles from one woman were excluded because the patient had been diagnosed with colorectal cancer 7 years prior to stimulation start, but had no active cancer at the time of stimulation. The remaining 147 cycles performed in 147 women were included in the final cancer patient dataset. A comparison group was identified, comprised of women undergoing their first ovarian stimulation cycle for IVF due to male factor infertility, with no evidence of female causes of infertility (*n* = 664). These women were selected because they represent presumably fertile women, thus enabling identification of any differences in ovarian stimulation outcomes exclusively attributable to cancer in the cancer patients.

### Exposure definitions

In primary analyses, women with cancer were grouped according to the distribution of their cancer as either local (stage I-III solid malignancy) or systemic (hematologic [i.e. leukemia or lymphoma] or stage IV solid malignancy). These patients were also classified by their cancer treatment prior to FP as having: 1) no chemotherapy; 2) exposure to any chemotherapy; or 3) exposure to tamoxifen or letrozole during stimulation. Women with systemic cancer were sub-classified as either having or not having exposure to chemotherapy or abdominal radiation prior to FP. Because there is evidence that BRCA carrier status may affect outcomes [6], women with cancer were also sub-classified as those with breast cancer who either were or were not carriers of BRCA gene mutations (either BRCA-1 or BRCA-2), or who were diagnosed with other cancers. Women with breast cancer without known BRCA-carrier status were excluded from this analysis, as was one woman who was diagnosed with both breast and thyroid cancer.

### Outcomes of interest

Age, baseline antral follicle count (AFC), and anti-Müllerian hormone (AMH) levels were assessed for all women, as were starting and total follicle stimulating hormone (FSH) doses, serum estradiol levels at time of ovulatory trigger, the duration of stimulation, total follicle number at ovulation trigger, total number of oocytes and number of mature oocytes retrieved, number of two pronucleate (2PN) embryos obtained, and whether or not the cycle was cancelled.

As there is often insufficient time for assessment of early follicular serum FSH and estradiol levels in women with cancer, these were not universally performed. Menstrual cycle phase at the start of stimulation was recorded, in addition to documentation of the exact cancer diagnoses, the BRCA mutation carrier status for women with breast cancer, and the types of cancer treatments each patient underwent prior to stimulation. For women who returned to use their frozen eggs and/or embryos in subsequent cycles, the duration of egg/embryo freezing and use of a gestational carrier were noted, as were the outcomes of the transfers, including pregnancy results, gestational age at delivery, and birth weights.

### Stimulation protocol and retrievals

All women undergoing fertility preservation were extensively counseled by an interdisciplinary team about the risks and benefits of undergoing ovarian stimulation either before or after their cancer treatment, including the potential risk of birth defects in offspring in patient recently exposed to systemic therapy.

Women with no cancer were stimulated during the early follicular phase of their menstrual cycles, as is conventional. Due to the time-sensitive nature of cancer treatments, women with cancer were stimulated either during the early follicular phase or at another random point in their menstrual cycles.

Several different types of protocols were used for ovarian stimulation; none were excluded so as to capture the full range of patients who may be undergoing fertility preservation. Furthermore, randomized trials have not found that IVF outcomes differ between agonist and antagonist cycles. The reasons for the protocols chosen were not explicitly discussed in each patient’s chart and likely represent provider preference. Gonadotropin releasing hormone (GnRH) antagonist protocols involved therapy with cetrorelix or ganirelix acetate (0.25 mg/d starting on stimulation day 6, and in some cases preceded by 5–21 days of oral contraceptive [OC] pills). Down-regulation protocols included: [1] “low dose” luteal phase down-regulation with leuprolide acetate (LA; 0.5 mg/d from cycle day 21 to 2 days after menses followed by a reduction of LA to 0.25 mg/d after gonadotropin administration began), [2] “very low dose” luteal phase down-regulation with LA (0.2 mg/d from cycle day 21 to 2 days after menses followed by a reduction of LA to 0.1 mg/d after gonadotropin administration). Poor-responder protocols included: [1] “micro dose” stimulation with LA (0.05 mg administered twice daily from cycle day 1 preceded by 7–21 days of OC pills); [2] “mini dose” luteal phase down-regulation with LA (0.5 mg/d from cycle day 21 to the day of gonadotropin initiation, then discontinued); [3] “ultra-low dose” luteal phase downregulation with 0.05 mg LA dropping down to 0.025 mg; and [4] a “estrogen priming” protocol with GnRH antagonist and transdermal estradiol starting 10 days after the prior cycle LH surge until the following cycle day 2 when stimulation began.

Women with estrogen-receptor positive breast cancers were offered letrozole or tamoxifen as adjunct medications during the stimulation cycle to reduce the theoretical risk stemming from increased estrogen exposure, as previously described [7, 8].

The majority of cancer patients underwent GnRH antagonist protocols (*n* = 139). For those undergoing conventional stimulation in the early follicular phase, recombinant FSH with or without human menopausal gonadotropin was started on cycle day 2 when possible. For patients undergoing a random stimulation start, exogenous gonadotropins were administered at any time during the menstrual cycle. Follicular development in all patients was monitored (assessing for follicles > 12 mm) as is standard and hCG or leuprolide was administered for final follicular maturation 36 h prior to oocyte retrieval when at least 2 follicles reached a mean diameter of 18 mm. Oocyte retrievals were performed transvaginally under ultrasound guidance.

Oocytes and embryos were cryopreserved either via slow cooling (prior to June 2012, using protocols as described previously, [9]) or with vitrification (from June 2012 on, using the Irvine Scientific protocol and HSV device [Irvine Scientific, Irvine, CA]). For those cancer patients planning to freeze embryos, oocytes were fertilized using standard IVF or ICSI as indicated and all embryos were cryopreserved at the 2PN stage.

### Statistical analysis

Multivariable linear regression was used to calculate β-coefficients with 95% confidence intervals for starting and total FSH doses, and serum estradiol levels at the time of ovulation trigger. Poisson regression was used to calculate relative risks for baseline AFC, duration of stimulation, total follicle number at hCG trigger, the total number of oocytes and the number of mature oocytes retrieved, proportion of mature oocytes retrieved, the oocyte/AFC ratio, mature oocyte/AFC ratio, and the number of embryos created. Of note, all stimulation protocol types were included in the analyses to minimize confounding by indication. Logistic regression was used to calculate the odds of a woman experiencing cycle cancellation. These analyses were adjusted for the woman’s age and body mass index (BMI) at the start of stimulation. As appropriate, the calculation for the number of embryos created was also adjusted for the use of ICSI. We did not adjust for gonadotropin doses because gonadotropins lie on the causal pathway between the exposure (cancer diagnosis) and the outcomes of interest (e.g. oocyte yield). A Wald *p*-value of less than 0.05 was considered to be significant throughout. The SAS statistical software version 9.3 was used for all analyses (SAS Institute Inc., Cary, NC).

## Results

The baseline characteristics of women included in our study population are presented in Table 1. Women with systemic cancer were younger (27.1 ± 6.4y) than those with either local cancer (33.6 ± 4.8y) or no cancer (34.6 ± 4.2y), but they had lower AMH levels (2.0 ± 2.2 ng/mL) than women with local cancer (2.8 ± 2.7 ng/ml) or no cancer (3.4 ± 3.3 ng/ml). As previously stated, the majority of women with no cancer were stimulated via down-regulation protocols, while most women with systemic or local cancer were stimulated using GnRH antagonist protocols. Furthermore, not all women with cancer started stimulation in the early follicular phase. Specifically, 21.7% of all cancer patients, 40.5% of those with systemic cancer and 14.3% of those with local cancer, were stimulated at a random time during their menstrual cycles.

**Table 1:** Baseline characteristics of women without cancer and women with local or systemic cancer

Characteristics at cycle start	No Cancer (*n* = 664)	Local Cancer (*n* = 105)	Systemic Cancer (*n* = 42)
Woman’s Age at Stimulation, years (mean ± SD)	34.6 ± 4.2	33.6 ± 4.8	27.1 ± 6.4
Baseline Antral Follicle Count (mean ± SD) (missing, *n* = 48)	9.4 ± 7.2	10.1 ± 7.4	7.3 ± 7.5
AMH, ng/mL (mean ± SD)	3.4 ± 3.3	2.8 ± 2.7	2.0 ± 2.2
Cycle Day 2–4 FSH, mIU/mL (mean ± SD) (missing, *n* = 91)	7.2 ± 2.3	9.6 ± 19.8	9.1 ± 6.3
Woman’s BMI at Stimulation, kg/m^2^ (mean ± SD)	25.7 ± 4.2	26.0 ± 6.3	24.9 ± 5.9
Woman’s Race (n [%])			
Caucasian	466 (70.2%)	88 (86.2%)	36 (85.7%)
Other	198 (29.8%)	14 (13.7%)	6 (14.3%)
Current Smoker (n [%])	17 (2.6%)	6 (5.7%)	0 (0%)
Gravida (n [%])	186 (28.3%)	35(33.3%)	7 (16.7%)
Cycle Type (n [%])			
GnRH antagonist	88 (13.3%)	103 (98.1%)	36 (85.7%)
Down-regulation	533 (80.3%)	1 (1.0%)	4 (9.5%)
Gonadotropin only	3 (0.5%)	1 (1.0%)	2 (4.8%)
Poor-responder protocols	40 (6.0%)	0 (0%)	0 (0%)
Start of Stimulation (n [%])			
Conventional/Early Follicular	664 (100.0%)	84 (80.0%)	25 (59.5%)
Random Start	0 (0%)	16 (14.3%)	17 (40.5%)
Late Follicular	0 (0%)	3 (2.9%)	2 (4.8%)
Luteal	0 (0%)	7 (6.7%)	5 (11.9%)
Total Motile Post-wash Sperm Count, 10^6^ (mean ± SD)	5.66 ± 16.88	28.9 ± 33.4	34.8 ± 41.5
ICSI (n [%])	547 (85.6%)	21 (21.7%)	4 (11.4%)

Table 2 shows the distribution of cancer patients regarding their cancer diagnoses. Approximately half had been diagnosed with breast cancer (53.7%), with 79.4% of these reporting BRCA negative tumors. One woman was diagnosed with both breast and thyroid cancer at the time of ovarian stimulation.

**Table 2:** Cancer diagnoses of women undergoing fertility preservation

Pre-therapy diagnosis	n (%)
Breast	79 (53.7)
Breast cancer type^a^	
Estrogen Receptor Positive	64 (81.0)
Estrogen Receptor Negative	15 (19.0)
Inflammatory	2 (2.5)
HER-2 Positive	18 (22.8)
BRCA status^b^	
Negative	51 (64.6)
BRCA-1 Positive	7 (8.9)
BRCA-2 Positive	6 (7.6)
BRCA-1 and − 2 Positive	0 (0.0)
Gynecologic	8 (5.4)
Ovarian	1 (0.7)
Endometrial	4 (2.7)
Cervical	5 (3.4)
Hematologic	38 (25.9)
Leukemia	11 (7.5)
Hodgkin’s lymphoma	18 (12.2)
Non-Hodgkin’s lymphoma	1 (0.7)
Myelodysplasia	2 (1.4)
Gastrointestinal	6 (4.1)
Brain	6 (4.1)
Other	11 (7.5)
Total	147^c^ (100)

^a^ Breast cancer type percentages reported as percent of all breast cancers

^b^ BRCA status percentages reported as percent of all breast cancers. Fifteen patients had an unknown BRCA mutation carrier status

^c^ One patient was diagnosed with both primary breast and thyroid (other) malignancy at the same time, and is thus only represented once in the Total row

### Effect of cancer

Comparisons of outcomes following ovarian stimulation for the three groups of patients are shown in Table 3. Women with systemic cancer had lower AFC at baseline, compared to those with either no cancer (*p* = 0.003) or local cancer (*p* = 0.04). Women with either local or systemic cancer were started on higher doses of FSH compared to women with no cancer (both *p* < 0.001); and they both required higher total doses of FSH (both p < 0.001). Women with systemic cancer received higher starting (p < 0.001) and total doses of FSH (*p* = 0.0031) than women with local cancer. Furthermore, women with systemic cancer had a greater odds of undergoing cycle cancellation as compared to women with no cancer (p < 0.001) or local cancer (*p* = 0.0016). Those with systemic cancer who did not undergo cycle cancellation had a lower total follicle number on the day of ovulatory trigger than women with no cancer; this was primarily driven by adjustment for age (*p* = 0.02). Women with systemic cancer also had higher proportion of eggs retrieved as compared to the AFC that cycle (oocyte to AFC ratio) than women with no cancer (*p* = 0.01) or local cancer (*p* = 0.04), as well as a higher mature oocyte to AFC ratio (*p* = 0.02 and *p* = 0.04, respectively). Notably, there were no significant differences among the three groups regarding duration of stimulation, the number of oocytes and mature oocytes retrieved, or the number of 2PN embryos obtained.

**Table 3:** Ovarian stimulation cycle outcomes among women without cancer and women with local or systemic cancer

Cycle characteristic	No Cancer (*n* = 664)	Local Cancer (*n* = 105)	Systemic Cancer (*n* = 42)
Woman’s Age at Stimulation (years)	34.6 ± 4.2	33.6 ± 4.8	27.1 ± 6.4
Baseline AFC (n)^1^	9.4 ± 7.2	10.1 ± 7.4	7.3 ± 7.5
	1.00 (Ref)	1.03 (0.89,1.21)	0.58 (0.41,0.83)*
		1.00 (Ref)	0.64 (0.42,0.97)*
Starting FSH dose (IU)^2^	289.0 ± 121.3	354.2 ± 170.6	417.8 ± 180.7
	0.00 (Ref)	80.9 (58.2103.6)*	252.4 (215.8289.1)*
		0.00 (Ref)	158.80 (95.7221.9)*
Total FSH dose (IU)^2^	1839.2 ± 1294.7	2813.6 ± 1785.6	3358.9 ± 2147.3
	0.00 (Ref)	1094.0 (825.9,1362.1)*	2483.0 (2050.8,2915.2)*
		0.00 (Ref)	1124.82 (380.5,1869.2)*
Duration of stimulation (days)^1^	11.7 ± 2.0	11.7 ± 2.3	12.2 ± 2.2
	1.00 (Ref)	1.00 (0.96,1.04)	1.06 (0.99,1.14)
		1.00 (Ref)	1.03 (0.94,1.12)
Total follicle number at hCG trigger (n)^1^	12.9 ± 6.6	12.2 ± 8.4	13.2 ± 5.90
	1.00 (Ref)	0.94 (0.85,1.04)	0.81 (0.68,0.96)*
		1.00 (Ref)	0.91 (0.75–1.12)
Number of oocytes retrieved (n)^1^	15.7 ± 8.6	16.8 ± 13.6	20.6 ± 21.0
	1.00 (Ref)	1.04 (0.89,1.21)	1.03 (0.68,1.55)
		1.00 (Ref)	1.05 (0.60,1.86)
Number of mature oocytes retrieved (n)^1^	12.0 ± 7.1	12.2 ± 8.4	16.0 ± 14.8
	1.00 (Ref)	0.99 (0.86,1.13)	1.02 (0.70,1.50)
		1.00 (Ref)	1.12 (0.68,1.84)
Proportion of mature oocytes (n/n)^1^	0.76 ± 0.19	0.76 ± 0.20	0.78 ± 0.15
	1.00 (Ref)	0.96 (0.81,1.14)	0.93 (0.60,1.42)
		1.00 (Ref)	0.94 (0.57,1.52)
Oocytes/AFC ratio (n/n)^1^	2.28 ± 2.87	2.06 ± 2.11	3.92 ± 7.35
	1.00 (Ref)	0.93 (0.67,1.28)	2.29 (1.01,5.23)*
		1.00 (Ref)	2.15 (0.86,5.40)
Mature oocytes/AFC ratio (n/n)^1^	1.76 ± 2.33	1.56 ± 1.59	2.82 ± 4.97
	1.00 (Ref)	0.90 (0.65,1.25)	2.09 (0.94,4.65)
		1.00 (Ref)	2.01 (0.81,4.99)
Number of embryos (n)^3^	8.9 ± 6.3	8.8 ± 6.4	12.3 ± 11.7
	1.00 (Ref)	0.93 (0.85,1.03)	1.03 (0.88,1.19)
		1.00 (Ref)	1.16 (0.98,1.39)
Cycle Cancelled [n (%)]^4^	14 ± 2.1	2 ± 1.9	9 ± 21.4
	1.00 (Ref)	0.92 (0.21,4.11)	14.41 (4.83,42.98)*
		1.00 (Ref)	17.03 (2.94,98.71)*

All results reported as mean ± standard deviation, unless otherwise noted

* indicates significance (*p*-value < 0.05)

^1^Poisson regression estimate, RR (95% CI), Adjusted for age and BMI at cycle start

^2^Linear regression estimate, β (95% CI), Adjusted for age and BMI at cycle start

^3^Poisson regression estimate, β (95% CI), Adjusted for age and BMI at cycle start, and ICSI use

^4^Logistic regression estimate, OR (95% CI), Adjusted for age and BMI at cycle start

### Effect of prior chemotherapy

Women with cancer who received chemotherapy prior to FP (*n* = 38) had lower baseline AFC than women with no cancer (4.7 ± 4.5 vs. 9.4 ± 7.2, *p* < 0.0001). These women were started on higher FSH doses (467.7 ± 160 vs. 289.0 ± 121.3 IU, *p* < 0.0001), and received higher total doses of FSH (4168.7 ± 160.0 vs. 1839.2 ± 1294.7 IU, *p* < 0.0001) over a longer stimulation (12.9 ± 2.5 vs. 11.7 ± 2.0 days, *p* = 0.002). They had a lower total follicle number at hCG trigger (11.5 ± 6.3 vs. 12.9 ± 6.6, *p* < 0.0001), had fewer oocytes (14.6 ± 9.3 vs. 15.7 ± 8.6, *p* = 0.001) and fewer mature oocytes retrieved (11.8 ± 7.7 vs. 12.0 ± 7.1, *p* = 0.004), with fewer embryos obtained (6.2 ± 6.7 vs. 8.9 ± 6.3, *p* = 0.0002). These women also had 22.2-fold greater odds of having a cycle cancellation than women with no cancer.

Women with cancer but without any chemotherapy before ovarian stimulation (*n* = 49) were also started on higher doses of FSH (348.2 ± 163.9 IU vs. 289.0 ± 121.3 IU, *p* < 0.0001) and required significantly higher total FSH doses (2508.7 ± 1697.5 IU vs. 1839.2 ± 1294.7 IU, *p* < 0.0001) than women with no cancer. The two groups did not differ in baseline AFC, total follicle number at hCG trigger, or the total number of oocytes or mature oocytes retrieved. Similar results were observed for the subset of women with cancer who were chemotherapy naïve, but who received either tamoxifen or letrozole as adjunct medications during their cycles (*n* = 60). Notably, the serum estradiol levels for women who had not been exposed to chemotherapy (1824.8 ± 1091.6 pg/mL, *p* = 0.001) and who had received prior chemotherapy (1344.8 ± 685.1 pg/mL, *p* < 0.0001) were lower than for women with no cancer (2229.0 ± 905.7 pg/mL). In sensitivity analyses, after restriction to letrozole-treated cases only, similar trends were observed (812.9 ± 600.0 pg/mL, *p* < 0.0001).

Women with systemic cancer who underwent any chemotherapy or abdominal radiation prior to FP had lower baseline AFC compared with those with no cancer (*p* < 0.0001). These women were started on higher doses of FSH (*p* < 0.001) and received higher total doses of FSH (*p* < 0.0001) over a longer stimulation (*p* = 0.003) than women with no cancer. Moreover, they had greater odds of cycle cancellation (*p* < 0.0001), lower total follicle numbers at hCG trigger (*p* = 0.0007), had fewer oocyte (*p* = 0.01) and mature oocytes (*p* = 0.02) retrieved, as well as fewer embryos (*p* = 0.0008) obtained compared to women with no cancer. The oocyte (*p* = 0.01) and mature oocyte to AFC ratio (*p* = 0.006) in these women was also higher than in women with no cancer. Those who had not been exposed to any chemotherapy or abdominal radiation prior to FP, were started on higher doses of FSH (*p* = 0.001) than the women with no cancer. However, their total FSH dose was not significantly greater than women with no cancer and, interestingly, they had a significantly higher number of oocytes and mature oocytes retrieved (both *p* = 0.03), with more embryos obtained (*p* < 0.0001).

Patients with systemic cancer who had undergone chemotherapy or abdominal radiation prior to fertility preservation were stimulated more aggressively, yet exhibited a poorer response to ovarian stimulation when compared to cancer patients who had not undergone any cancer treatment prior to cycle start. Higher starting (*p* = 0.002) and total doses of FSH (*p* = 0.0004) were used, fewer total follicles at hCG trigger (*p* = 0.01) were observed, fewer total oocytes (*p* = 0.0004) and mature oocytes (*p* = 0.0010) were retrieved, and fewer embryos were obtained (*p* < 0.0001).

The effect of time elapsed between prior chemotherapy and/or radiation and ovarian stimulation on fertility preservation outcomes was not explored due to the variable durations of treatment.

### Effect of menstrual cycle phase

Compared to women with no cancer, whether women with cancer were stimulated in the early follicular phase (*n* = 109) or at a random time of their menstrual cycle (*n* = 33), they used higher starting doses of FSH (288.26 ± 120.72 IU vs. 383.6 ± 175.4 and 319.3 ± 162.5 IU, respectively) and higher total doses of FSH (1835.0 ± 1293.3 IU vs. 2965.6 ± 1961.7 and 2727.27 ± 1617.2 IU, respectively). Those who underwent random-start stimulation tended towards lower starting and total FSH doses than those with cancer who underwent early follicular stimulation, and they had greater odds of having a cycle cancellation compared to women with no cancer [OR: 6.95 (2.16–22.38), *p* < 0.05]. Of the five women who underwent random-start stimulation and had cycle cancellation, all had received prior chemotherapy (four of them alkylating agents) and three of them had AMH levels of 0.3 (levels were not available for the other two patients). This suggests that cycle cancellation was a function of decreased ovarian reserve, rather than the effect of random start stimulation. Furthermore, the random start group had more oocytes (23.0 ± 18.9) and mature oocytes (16.1 ± 9.5) retrieved than either women with no cancer (15.7 ± 8.6 and 12.0 ± 7.1, respectively) or those with cancer who underwent early follicular stimulation (16.6 ± 14.7 and 12.5 ± 10.6, respectively), although none of these differences reached significance. This pattern of association was consistent in an intention-to-treat analysis after inclusion of cancelled cycles in each group.

### Effect of breast cancer and BRCA mutation

Because breast cancer is the most common malignancy in women of reproductive age [10], we investigated any association between the presence of breast cancer, as well as BRCA mutation on the outcomes of ovarian stimulation (Table 4). Women with breast cancer were started on significantly higher doses of FSH and received significantly higher total units of FSH than those with no cancer. Furthermore, women with breast cancer who were BRCA-negative had significantly fewer mature oocytes retrieved than women with no cancer, with fewer zygotes created. These differences were not seen in women with breast cancer who were carriers of BRCA 1 or 2 mutations, however we had limited power (*n* = 13) to detect associations.

**Table 4:** Cycle outcomes among women with no cancer, breast cancer +/− BRCA mutations, or other cancer

Cycle characteristics	No Cancer (*n* = 664)	BRCA- Breast Cancer (*n* = 49)	BRCA+ Breast Cancer (*n* = 13)	Other Cancer (*n* = 68)
Woman’s Age at Stimulation (years)	34.6 ± 4.2	34.7 ± 3.7	32.3 ± 4.0	28.5 ± 6.4
Baseline AFC (n)^1^	9.4 ± 7.2	11.5 ± 7.7	7.7 ± 6.3	8.3 ± 7.8
	1.00 (Ref)	1.22 (0.99,1.50)	0.76 (0.50,1.15)	0.70 (0.54,0.90)*
Starting FSH dose (IU)^2^	289.0 ± 121.3	342.0 ± 159.6	395.2 ± 236.5	375.5 ± 174.5
	0.0 (Ref)	53.5 (21.1,85.9)*	142.8 (81.5204.1)*	183.2 (153.3213.1)*
Total FSH dose (IU)^2^	1839.2 ± 1294.7	2498.0 ± 1570.9	4326.9 ± 3046.6	2994.5 ± 1715.8
	0.00 (Ref)	637.7 (302.4,1044.9)*	2775.0 (2072.0,3478.0)*	1916.5 (1573.7,2259.4)*
Duration of stimulation (days)^1^	11.7 ± 2.0	11.3 ± 2.4	13.0 ± 3.0	12.1 ± 2.0
	1.00 (Ref)	0.96 (0.91,1.02)	1.12 (0.98,1.28)	1.05 (1.00,1.10)*
Total follicle number at hCG trigger (n)^1^	12.9 ± 6.6	12.1 ± 5.8	12.1 ± 5.5	13.3 ± 5.9
	1.00 (Ref)	0.95 (0.83,1.09)	0.87 (0.68,1.12)	0.85 (0.75,0.97)*
Number of oocytes retrieved (n)^1^	15.7 ± 8.6	14.9 ± 9.6	15.5 ± 7.1	20.8 ± 19.7
	1.00 (Ref)	0.94 (0.78,1.12)	0.86 (0.67,1.12)	1.11 (0.84,1.45)
Number of mature oocytes retrieved (n)^1^	12.0 ± 7.1	10.2 ± 5.9	11.5 ± 5.6	15.7 ± 12.4
	1.00 (Ref)	0.85 (0.72,0.99)*	0.90 (0.69,1.16)	1.08 (0.85,1.38)
Proportion of mature oocytes (n/n)^1^	0.76 ± 0.19	0.78 ± 0.21	0.79 ± 0.07	0.80 ± 0.16
	1.00 (Ref)	0.97 (0.78,1.19)	1.23 (0.94,1.60)	0.94 (0.69,1.26)
Oocytes/AFC ratio (n/n)^1^	2.28 ± 2.87	1.85 ± 2.15	2.29 ± 1.25	3.19 ± 5.71
	1.00 (Ref)	0.73 (0.46,1.18)	1.37 (0.66,2.82)	1.69 (0.95,2.98)
Mature oocytes/AFC ratio (n/n)^1^	1.76 ± 2.33	1.29 ± 1.43	1.77 ± 0.94	2.39 ± 3.93
	1.00 (Ref)	0.66 (0.41,1.06)	1.37 (0.66,2.80)	1.62 (0.94,2.80)
Number of embryos (n)^3^	8.9 ± 6.3	8.0 ± 10.0	6.4 ± 5.7	6.2 ± 6.7
	1.00 (Ref)	0.84 (0.74,0.97)*	0.90 (0.67,1.21)	1.09 (0.98,1.23)
Cycle cancelled [n (%)]^4^	14 (2.1)	0 (0)	2 (15.4)	8 (11.8)
	1.00 (Ref)	–	8.55 (1.70,42.88)*	6.06 (2.12,17.35)*

All results reported as mean ± standard deviation, unless otherwise noted

* indicates statistical significance (*p*-value < 0.05)

^1^Poisson regression estimate, RR (95% CI), Adjusted for age and BMI at cycle start

^2^Linear regression estimate, β (95% CI), Adjusted for age and BMI at cycle start

^3^Poisson regression estimate, β (95% CI), Adjusted for age and BMI at cycle start, and ICSI use

^4^Logistic regression estimate, OR (95% CI), Adjusted for age and BMI at cycle start

### Cryopreservation and pregnancy outcomes

**Fig. 1:**
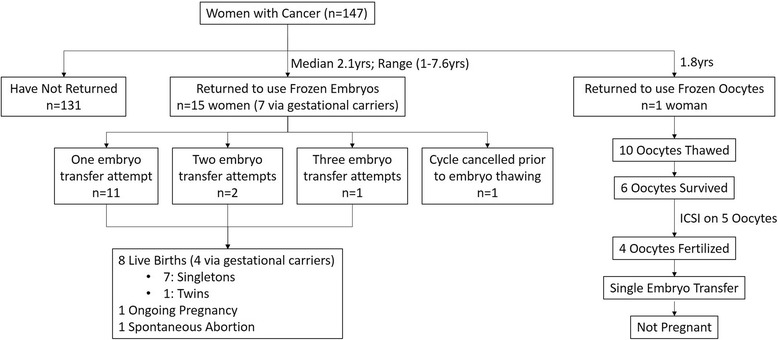
Cycle outcomes among women with cancer who returned to use their cryopreserved embryos or oocytes

All women with cancer who had oocytes retrieved and/or embryos created elected to have cryopreservation. To date, 19 of the 147 cancer patients are now deceased. One of the 147 women returned to use her frozen oocytes, which resulted in one transfer, and 15 returned to use their frozen embryos for a total of 18 transfers (see Fig. 1). None of the transfer cycles employed preimplantation genetic screening (PGS), as we do not recommend PGS in women with cancer due to many embryos being mosaic and potentially resulting in a live birth. Eight women had breast cancer, two had cervical cancer, and one each had chronic myelogenous leukemia, myelodysplastic syndrome, endometrial cancer with endometrioid ovarian cancer, recurrent liposarcoma, and lung cancer. The live birth rates were 40.0% per cycle start (8 of 20 cycles), 42.1% per embryo transfer (8 of 19), and 50.0% per woman treated (8 of 16). Seven deliveries resulted in singleton births (gestational age at delivery ranging from 38 + 6/7 to 41 + 2/7 weeks), and one resulted in a set of twins (gestational age, 35 + 6/7 weeks). Birth weights ranged from 2381 to 4706 g with an average of 3478 g. One of the patients who had a singleton via gestational carrier died soon after delivery; this had been anticipated and extensive medical, social, and ethics consults were obtained prior to the cryopreserved embryo transfer cycle. One pregnancy was ongoing at the time of writing and one pregnancy resulted in a spontaneous abortion.

## Discussion

In this study, we compared ovarian response to gonadotropins and cycle outcomes of women diagnosed with cancer who underwent IVF for purposes of FP to those of healthy, presumably fertile women whose partners had male factor infertility. Our data show that the numbers of oocytes and embryos obtained for women with cancer are equivalent to those for women with no cancer, particularly when the ovarian stimulation is performed prior to chemotherapy. However, women with cancer require higher doses of gonadotropins to achieve those yields. Compared with those with local cancer, women with systemic cancer and prior chemotherapy exposure are at greater risk of cycle cancellation. For the few patients who have returned to use their cryopreserved oocytes or embryos, the overall delivery rate was 40.0% per cycle start and 42.1% per embryo transfer. To our knowledge, this is one of the largest cohort studies to investigate the effect of cancer on FP outcomes.

Our data showed that women with local cancer had similar AFC at baseline as those with no cancer. These results are consistent with prior studies showing no difference in the baseline AFC between chemotherapy-naïve women with cancer and women with no cancer [11, 12]. However, women with systemic cancer, and specifically those with prior chemotherapy or abdominal radiation exposure, had significantly lower baseline AFC, demonstrating that chemotherapy and abdominal radiation are often gonadotoxic. This is also consistent with a recent study that found women with lymphoma have lower baseline AFC even prior to chemotherapy as compared to women with no cancer or other cancers [13].

With regard to stimulation characteristics, women with cancer were started on significantly higher doses of FSH than women with no cancer. The higher starting doses may suggest an underlying trend among providers treating all women with cancer, regardless of baseline tests of ovarian function and reserve. In the sub-population of patients with systemic cancer and prior chemotherapy or abdominal radiation exposure, the higher starting doses are likely the result of clinicians’ appropriate response to lower baseline AFC. Women with local cancer and women with systemic cancer and prior chemotherapy/radiation exposure subsequently received higher total doses of FSH than women with no cancer. In contrast, previous studies demonstrated no significant difference in the total dose of gonadotropins needed to stimulate follicular development in women with cancer [5, 11–17]. These results are particularly noteworthy since the GnRH antagonist protocol, which is the one most commonly used in this cohort, typically requires lower total FSH dosing than other protocols such as downregulation [18].

Nevertheless, it seems that higher FSH dosing can overcome the decreased ovarian responsiveness, as we found that women with local cancer had similar oocyte yields to women with no cancer. In women with systemic cancer and prior chemotherapy/radiation exposure, the higher total FSH doses resulted in a significantly higher oocyte to AFC ratio than women with no cancer, perhaps indicating better follicular recruitment. Thus, our results add to a conflicting literature, in which some previous studies reported significantly lower oocyte yields [13, 16, 19] and reduced fertilization rates in women with cancer [12], while others found no difference in the number of oocytes retrieved from women with cancer [5, 12, 14, 15, 20, 21].

Taken together, these observations suggest a similar adverse effect of cancer on gonadal function in women as previously reported for men diagnosed with advanced stage or systemic cancers. Several studies reported testicular dysfunction and semen abnormalities in patients with Hodgkin’s lymphoma [22, 23]. Interestingly, one study showed that the decreased fertility was most significant in the setting of elevated erythrocyte sedimentation rate (ESR) and advanced-stage disease, suggesting that systemic inflammation may interfere with gonadal function [24]. Unfortunately, we did not have access to ESR or C-reactive protein levels in most of the patients in our study cohort and so are unable to comment on any relationship between levels of these inflammation markers and ovarian response.

Our data suggest that in our cancer patients, random-start stimulation tended to require lower total doses of gonadotropins and, interestingly, resulted in a higher number of oocytes retrieved and embryos obtained than conventional stimulation in the early follicular phase. This conflicts with a prior study that showed that women with cancer undergoing random-start cycles required higher total doses of gonadotropins over a longer stimulation than women undergoing conventional starts, although the oocyte and embryos yields were no different [25]. Nevertheless, our results suggest that random-start stimulation is effective for urgent FP.

Our results in breast cancer patients suggest that BRCA-positivity does not have a negative impact on oocyte yield, although higher FSH dose requirements do suggest lower ovarian response among these patients as compared to patients with BRCA-negative tumors. This adds to already conflicting literature in which one study found significantly lower oocyte yields in women with BRCA-positive breast cancers than women with BRCA-negative breast cancer [6], while another showed no significant differences in oocyte or embryo yields [26].

The current study is one of the few to evaluate pregnancy rates after FP. We found a 42.1% delivery rate per transfer, which we consider good for day 3 embryo transfer in the setting of these patients having had all, not just good quality, embryos frozen and subsequently thawed and transferred. This rate is also consistent with our prior reports as well as those of others, which range from 12 to 75% [5, 12, 15, 27–32]. However, all studies, including our own, have small sample sizes ranging from 4 to 33 patients. Follow-up studies are planned as more patients return to use their cryopreserved oocytes and/or embryos.

The current study has several strengths. As one of the largest studies undertaken to examine ovarian stimulation outcomes for purposes of FP in women with malignancy, we had the statistical power to compare cycle outcomes of healthy women with male factor infertility to those of women with different types and stages of cancers. Moreover, we were able to perform subgroup analyses, otherwise not feasible with smaller patient populations. While several studies have attempted to stratify women with cancer by type (e.g. hematologic, breast, gastrointestinal tract, etc.), group sizes were small in all but one study [13], limiting interpretation of the data [11, 17, 20]. Furthermore, most published studies excluded women with cancer who had been exposed to chemotherapy; thus, knowledge is sparse regarding ovarian response after chemotherapy. However, in reality, cancer treatment cannot always be delayed (e.g. in cases of acute leukemia), and ovarian stimulation cannot always be started prior to chemotherapy. The findings in our study will help fill these knowledge gaps and will be valuable when counseling patients regarding expectations as they pursue FP.

## Conclusion

In conclusion, the findings of this study suggest that women with cancer undergoing FP achieve similar oocyte and embryo yields as women with no cancer, although those with systemic cancer and those exposed to prior chemotherapy or abdominal radiation require higher FSH doses and are at greater risk of cycle cancellation. Further studies are needed to explore the biological effect of deleterious BRCA mutations on ovarian response and whether aggressive stimulation protocols are needed for these patients. Long term follow-up studies of oocyte and embryo utilization are also needed as a greater number of women take advantage of FP. Taken together, our findings contribute to available evidence that 1) FP should be offered to pre-menopausal women diagnosed with cancer; and 2) even after chemotherapy, ovarian stimulation may yield oocytes although compared with non-cancer patients, higher gonadotropin doses may be needed and cycle cancellation risk is increased.

## Abbreviations

2PN: Two pronucleate embryos
AFC: Antral follicle count
AMH: Anti-Müllerian hormone
BMI: Body mass index
ESR: Erythrocyte sedimentation rate
FP: Fertility preservation
FSH: Follicle-stimulating hormone
GnRH: Gonadotropin releasing hormone
hCG: Human chorionic gonadotropin
ICSI: Intracytoplasmic sperm injection
IVF: In vitro fertilization
LA: Leuprolide acetate
LH: Luteinizing hormone
OC: Oral contraceptive
